# Stochastic Species Turnover and Stable Coexistence in a Species-Rich, Fire-Prone Plant Community

**DOI:** 10.1371/journal.pone.0000938

**Published:** 2007-09-26

**Authors:** Wilfried Thuiller, Jasper A. Slingsby, Sean D. J. Privett, Richard M. Cowling

**Affiliations:** 1 Laboratoire d'Ecologie Alpine, CNRS-UMR 5553, Université Joseph Fourier, Grenoble, France; 2 Department of Botany, University of Cape Town, Rondebosch, South Africa; 3 Department of Botany, Nelson Mandela Metropolitan University, Port Elizabeth, South Africa; Centre National de la Recherche Scientifique, France

## Abstract

Understanding the mechanisms that maintain diversity is important for managing ecosystems for species persistence. Here we used a long-term data set to understand mechanisms of coexistence at the local and regional scales in the Cape Floristic Region, a global hotspot of plant diversity. We used a dataset comprising 81 monitoring sites, sampled in 1966 and again in 1996, and containing 422 species for which growth form, regeneration mode, dispersal distance and abundances at both the local (site) and meta-community scales are known. We found that species presence and abundance were stable at the meta-community scale over the 30 year period but highly unstable at the local scale, and were not influenced by species' biological attributes. Moreover, rare species were no more likely to go extinct at the local scale than common species, and that alpha diversity in local communities was strongly influenced by habitat. We conclude that stochastic environmental fluctuations associated with recurrent fire buffer populations from extinction, thereby ensuring stable coexistence at the meta-community scale by creating a “neutral-like” pattern maintained by niche-differentiation.

## Introduction

Ecologists have always been intrigued by how species coexist [1], especially in species-rich communities [2]. While a great deal of research on patterns and mechanisms of coexistence is stimulated by curiosity about nature, understanding these mechanisms is also important for ecosystem management [3]. In this regard, heuristic and parsimonious models that assume biological neutrality [4] may not be a true reflection of reality and can thus be misleading or meaningless to ecological managers who require guidelines that are underpinned by theories and models that are useful in practice [5].

Theoretical and empirical research has demonstrated the key role of environmental variability in maintaining species richness in plant communities [6]–[9]. In many cases, environmental variability stabilises coexistence as a consequence of differential, species-specific responses to fluctuations in resources and opportunities for recruitment (e.g. [6], [10]). A potent form of environmental variation is associated with variation in fire regime components (frequency, season and intensity), which significantly influences the composition and diversity of plant communities at the local scale [11]. This is especially true of the Mediterranean shrublands of South Africa (fynbos) and Australia (kwongan) [12] that support landscapes which, after the wet tropics, are the most species-rich in the world [13].

Fynbos–a fire-prone, sclerophyllous shrubland that grows on nutrient-poor sands-is the dominant vegetation of South Africa's Cape Floristic Region, a globally recognised centre of plant diversity and endemism. This region is home to 9000 species, 69% of which are endemic [14]. At the meta-community scale, diversity is exceptionally high, especially of range-restricted rare species, many of which occur in small and isolated populations [15]. A major challenge for management is to minimize the risks of extinction of species, a difficult task in the face of habitat fragmentation, invasive alien plants, and altered fire and hydrological regimes [16]. In order to manage for species' persistence, fynbos managers require an understanding of the mechanisms that maintain diversity and the factors that may alter these mechanisms.

Fire is the principal source of disturbance in fynbos, and fire regimes vary greatly in their season of occurrence, frequency, extent and intensity [11]. Species respond differently to environmental fluctuations associated with stochastic variation in fire regime components and post-fire environments (e.g. [17], [18]), thereby creating large population fluctuations, some of which produce local extinctions. Fire-induced, differential population responses of species–for example hot fires favouring the germination of large-seeded species and cool fires favouring small-seeded ones [17], [19], or frequent fires favouring rapidly maturing species and infrequent fires favouring slow maturing ones [20]–result in compositional changes over time that may shift competitive hierarchies and, hence, mediate long-term coexistence [21]. This is a niche-based model of coexistence as it requires that species have different optimal conditions for growth and recruitment (i.e. segregation along a niche axis, e.g. [22]). Should environmental fluctuations be altered in such a way that particular conditions occur less often or not at all–for example increased fire intensity because of increased fuel loads as a result of invasive alien species [23], [24], or increased fire frequency as the result of anthropogenic ignition sources [25] or climate change ([26], [27])–then the processes mediating coexistence may no longer operate effectively, resulting in local and global species' extinctions.

We hypothesize that stochastic variability in fire regimes and post-fire weather conditions would promote coexistence in fynbos communities at local as well as at larger (metacommunity) scales via a temporal storage effect [7], [9], [28]. In other words, owing to differences in regeneration and resource niches, and the consequent differential responses of populations of component species to the conditions associated with a particular fire and the ensuing weather conditions, each species would somewhere, and at some time, experience population growth or decline [21]. At the metacommunity scale, the populations of species would show relatively stability, leading to stable coexistence. Testable implications of this hypothesis are (i) that population would remain stable over the same time period at the metacommunity scale; and (ii) that patterns of local-scale extinction and colonization over long time-scale encompassing several fire events would be equal across all species, irrespective of attributes such as local abundances, areas of occupancy and biological traits.

Here we investigated the mechanisms of coexistence at the local and regional scales in fynbos shrublands of South Africa's Table Mountain National Park, the most species-rich part of the Cape Floristic Region [29], [30]. Using two plant data sets (comprising 422 species) and collected in 1966 and 1996 from the same 81 monitoring sites in the Cape of Good Hope section of the park, we assessed patterns of species turnover, species spread and abundance at the local (monitoring site) and meta-community (entire protected area) scales. We tested for an equalizing mechanism [28] for coexistence at the local scale by modelling the relationships between the frequency of local extinctions and colonisations over the thirty-year period against biological attributes. To account for the taxonomic relatedness of species, we used generalised linear mixed models with taxonomy as random factors. More specifically, we addressed the following questions:

1. How does diversity vary between the two sampling dates?
2. Are species presence and abundance stable at both local and meta-community scales?
3. Are the odds of extinctions and colonisations at the local scale correlated with life history traits and other ecological or geographical attributes?

## Results

### Patterns of diversity

In 1966, 422 species were recorded in the 81 sites of the survey that we re-sampled in 1996, when we recorded 396 species. Of these, 339 (80.3%) were disappearers i.e. they were recorded as locally extinct from at least one of the sites that they occupied in 1966. A smaller tally–124 species (29.4%)–was not recorded at any site in the 1996 survey. Colonisers–species recorded somewhere in 1996 where they were not present in 1966–comprised 312 species or 73.9% of the 1996 tally. Of these, 62 species were not recorded anywhere in 1966. The 1966 sample included 65 families and 188 genera; corresponding data for the 1996 sample were 55 and 158.

**Figure 1:**
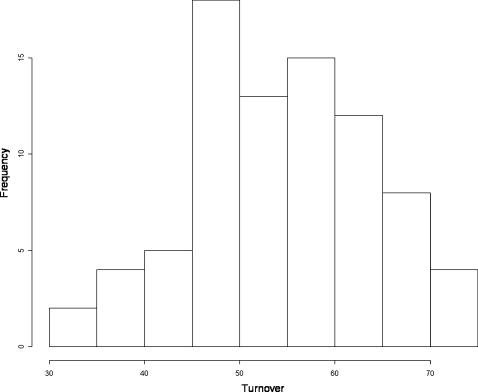
Frequency distribution of the temporal species turnover in the 81 sites sampled in 1966 and 1996.

Generally, at the local-scale, temporal species turnover was very high with 74% of sites experiencing >50% turnover between the two sampling dates (Fig. 1). The frequency distribution was moderately right-skewed indicating that only a few sites experienced a low turnover, and none having turnover <30%.

**Figure 2:**
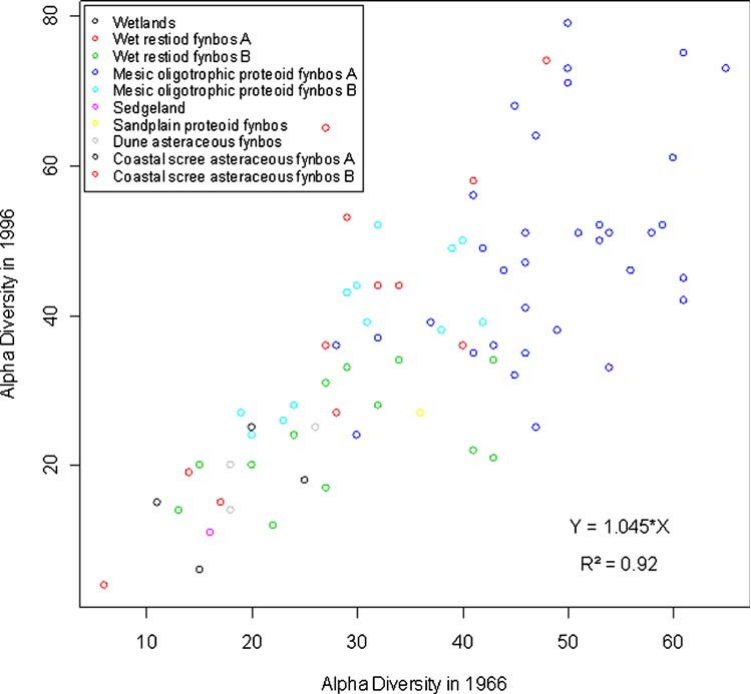
Relationship between alpha diversity measured in 1966 and in 1996.

Alpha diversity for both 1966 and 1996 was very similar (Fig. 2, *R^2^* = 0.92, *p*<0.001); therefore, the local diversity remained remarkably stable despite high turnover at the site scale. Similarly, beta similarity (Morisita-Horn measure) for 1966 and 1996 was highly correlated (Mantel-test *r* with 2000 permutations = 0.89), demonstrating that the community similarity between sites tended to remain stable at the meta-community scale (entire protected area) despite the high temporal turnover at the local scale. Despite massive changes in composition, local species diversity was largely determined by habitat since vegetation types were clearly segregated according to levels of local diversity at both sampling times. Interestingly wet restiod fynbos A spanned a wide range of alpha diversity values but the correspondence between the two sampling times remained strong, implying that there are well structured or distinct habitats within this vegetation type.

**Figure 3:**
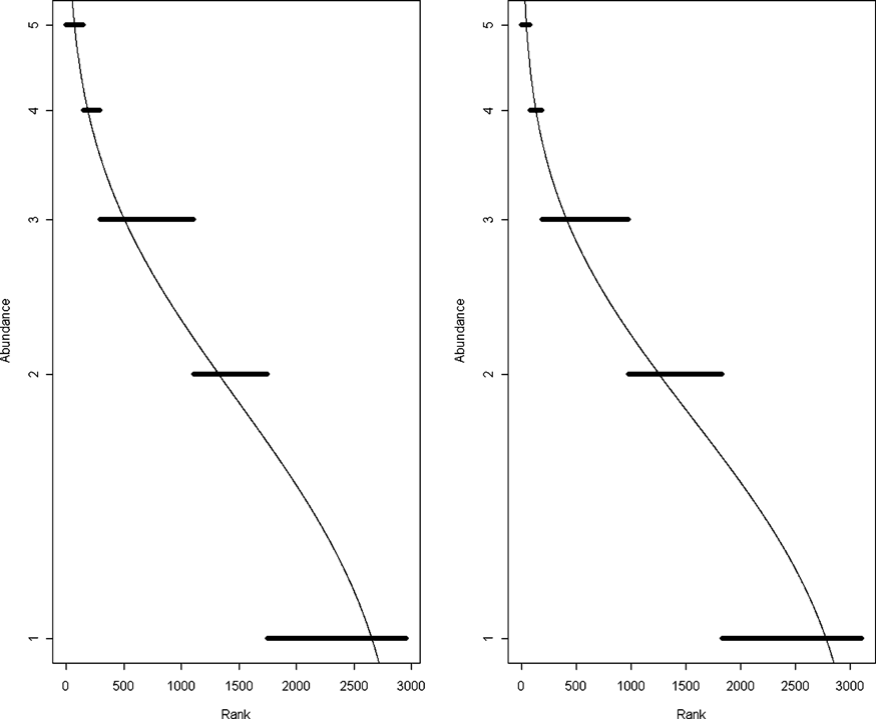
Dominance–diversity curves for both sampling dates (1966 and 1996). These plots display logarithmic species abundances against species rank order in the community [56].

Finally, despite the very high temporal turnover observed, the dominance–diversity curves for 1966 and 1996 were identical (Fig. 3), suggesting that temporal turnover may promote coexistence by avoiding competitive exclusion by the most dominant species.

Illustrative of this pattern is the case of *Leucadendron laureolum* (Proteaceae), a conspicuous and locally dominant non-sprouting, overstorey shrub. In 1966, this shrub occupied 36 sites. Corresponding data for 1996 were 37, indicating remarkable constancy at the meta-community scale. Patterns for this species are indicative of the remarkable turnover we observed at the site scale: *L. laureolum* disappeared from 10 sites and colonized 11 sites between 1966 and 1996.

### Patterns of spread and abundance at local and meta-community scales

**Figure 4:**
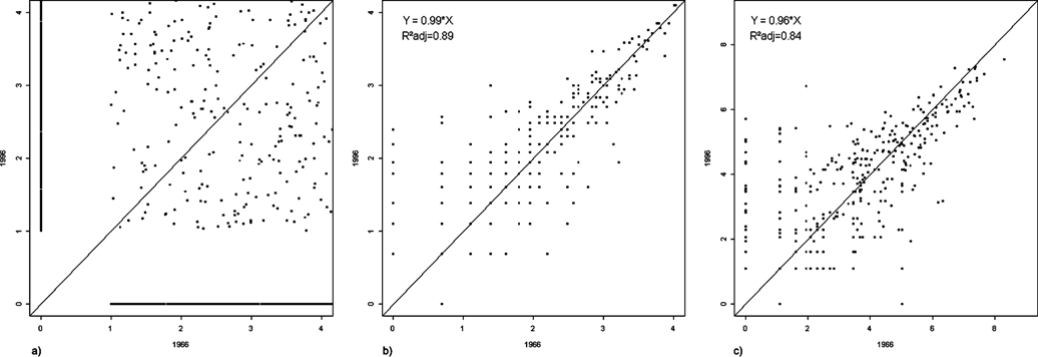
(a) Relationship between the local species abundance (populations of each species within each plot at the time considered) in 1966 and 1996. Data shown are for one set of estimated actual abundances, a range of *r^2^* values for 100 estimates are reported [*r^2^* = 0.037–0.058, p<0.001]. (b) Relationship between the meta-community spread (number of plots where a species is present) in 1966 and in 1996. (c) Relationship between the meta-community species abundance (total number of individuals for each species across all sites) in 1966 and 1996; The plots b and c are shown on a logarithmic scale.

There was no relationship between local species abundance (species' population sizes within each sample site) in 1966 and 1996 (Fig. 4a). The number of plots in which each species occurred (meta-community spread) and the total number of individuals per species (meta-community species abundance), however, showed strong and highly significant positive relationships between the two sampling periods (Figs 4b and 4c). This implies that despite the large temporal turnover at the local scale over the thirty-year interval (Fig. 1), and the instability of local population sizes (Fig. 4a), the spread and abundances of species at the meta-community scale was highly stable.

### Correlates of local extinctions and colonisations

**Figure 5:**
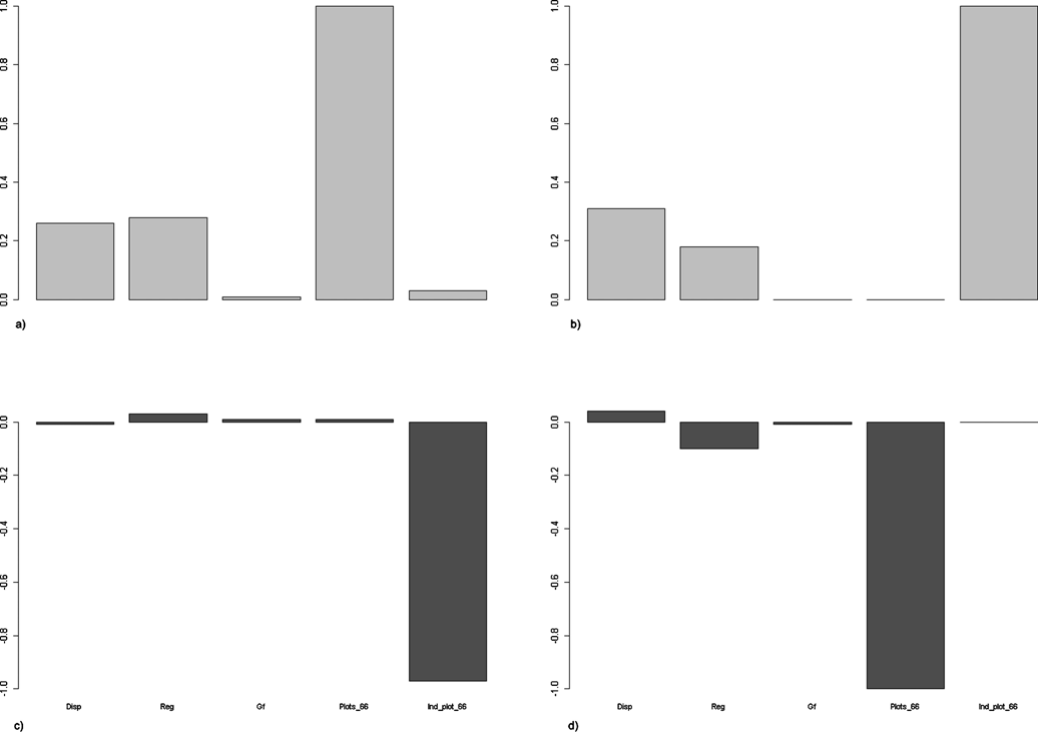
Relative (a and b, w*_pi_*) and absolute weights of evidence (c and d, Δw*_pi_*) of five derived predictors [dispersal model (Disp), regeneration type (Reg), growth form (Gf) and meta-community spread (Spread) and meta-community species abundance (Abundance) for explaining frequency of extinction (a and c) and frequency of colonisation (b and d). W*_pi_* are considered as relative, not absolute because they will be >0 even if the predictor has no explanatory importance due to random factors [49], [53]. Absolute weight of evidence from random permutation gives an unbiased estimation of the weight of evidence after accounting for random artefacts.

The local extinction and colonization frequencies modelled by generalised linear mixed models, were significantly, although poorly, correlated to the respective observed frequencies of extinction (*r* ^2^ = 0.50, *P*<0.0001) and colonization (*r* ^2^ = 0.29, *P*<0.0001) prior to accounting for random effects. This analysis revealed that meta-community spread (number of plots in which each species occurred in 1966) was the variable with the strongest relative weight of evidence to explain local patterns of extinctions, followed by dispersal distance and regeneration strategy (Fig. 5a). The local frequency of colonisation was best explained by the meta-community species abundance, once again followed by dispersal distance and regeneration strategy (Fig. 5b). Growth form was not an important explanatory variable in either case.

It is important to note that such weights of evidence could arise from random factors, or random stratifications of the data. Indeed, the re-sampling procedure that we performed in order to account for random processes showed that none of the explanatory variables had significantly positive absolute weights of evidence to explain either the local extinction or colonisation frequencies (Figs 5c and 5d). This demonstrates the utility of using re-sampling procedures to test for random effects.

In short, none of the biological or abundance attributes convincingly predicted the frequency of local extinction or colonisation in the study system.

## Discussion

The spread and abundance of individual species, and the patterns of alpha and beta diversity showed remarkable temporal constancy at the meta-community scale. This, as well as the identical dominance-diversity curves, implies that coexistence of species within the study area is relatively stable. Despite such high stability at the meta-community scale, our results indicate remarkably high temporal turnover of species at the local scale, a phenomenon that is confirmed by observations showing marked changes in the population sizes of individual fynbos species after particular fires [18], [31], [32]. We assume that most of this turnover is associated with the stochastic variability in fire regime effects (frequency, intensity, season) [11] and in the immediate post-fire weather conditions (wind, rainfall and temperature), all of which influence post-fire population sizes [18], [32]. However, shifting competitive hierarchies–for example the colonization of a site by an overstorey shrub such as *Leucadendron laureolum*–will also impact community composition as the vegetation matures, owing to species-specific negative and positive effects that this component has on the understorey flora [32], [33]. In the case of the nine sites that did not burn, turnover is likely to be associated with senescence of fast-maturing species and the recruitment of long-lived, bird-dispersed ones [34].

Species' abundance and biological attributes could not predict the local-scale extinction and colonization patterns. Surprisingly, the odds of sprouters becoming locally extinct were the same as non-sprouters, despite sprouters being widely regarded as representative of a persistence niche [35]. Although this implies that interactions between individuals, and interactions between individuals and the environment, are somewhat neutral, these results are consistent with our hypothesis that long-term patterns of local-scale extinction and colonization in response to one or more fire events would be equal across all species, irrespective of attributes such as local abundances, areas of occupancy and biological traits. Stochastic fire and post-fire weather effects act differentially on species recruitment at different times and places: the net result over the long term is species equivalence. Differential recruitment alone may represent the mechanism required for stable coexistence [21], whereby specific fires favour some species but cause the population reduction or even local extinction of others, as has been documented for *Leucadendron laureolum* in our study area [32].

The extent to which this stabilizing mechanism, which underscores the temporal storage effect [7], [28], is likely to be augmented by density dependent differences in the strength of intraspecific and interspecific interactions [31], [36], or density-dependent seed predation [37], remains to be assessed.. That species abundance cannot predict probabilities of local extinction implies that there is a rare species advantage (the invasibility criterion, [38]). This rare species advantage, or tendency of species to recover from low densities, is the fundamental requirement for stable species coexistence and implies that coexistence in our study site is mediated by a niche-based process such as a storage effect [28]. However, since fire induces differential population responses of species, and thus constantly shifts competitive hierarchies, stable coexistence may be mediated by a storage effect without having to invoke density-dependent interactions [21].

Populations in our study system may also be buffered by dispersal between sites, as local extinction of species is common, but colonization of local sites is equally common, resulting in a relatively stable distribution of species across the meta-community. This implies that the storage effect invoked here may be spatial as well as temporal in nature [2]. Coexistence would thus be mediated by species niche differences in relation to fire conditions. Each fire is unique in that different fires create different ‘habitats’, but habitats vary unpredictably in time due to stochasticity in the fire regime, thus preventing local community stability via competitor dominance or deterministic succession dynamics [23]. Species' permanence in the meta-community is therefore determined by source-sink dynamics between patches, but fire driven spatio-temporal variation in the suitability of patches prevents dominance by superior competitors and thus allows the coexistence of large numbers of species. Under this model species permanence and species coexistence are likely to be very sensitive to directional shifts in fire regime effects or post fire weather conditions.

Most importantly, we demonstrate stable coexistence at the metacommunity scale whereas in neutral community models, coexistence is unstable [39], [40]. Our long-term data on the turnover and abundance of species in fynbos demonstrate that stochastic environmental fluctuations associated with recurrent fire buffer populations from local extinction, thereby ensuring stable coexistence at the metacommunity scale. Data from a wide range of systems, namely desert annuals [8], prairie grasses [6] and coral reefs [41] are consistent with this model.

Finally, our coexistence model reinforces the need to introduce variation in fire regimes applied to fynbos ecosystems in order to maintain meta-community diversity [16]. The application of fixed regimes (e.g. autumn fires at 15-year intervals) is likely to repeatedly promote good recruitment of only a subset of the local community, resulting in species-impoverishment. Although post-fire weather conditions will vary stochastically even within particular seasons, the intensity of a fire-as determined by fuel loads and prevailing weather conditions–is likely to show much less variation if a fixed regime is applied [42]. Consequently, environmental variation should be maximized by introducing stochasticity into the application of management fires.

## Materials and Methods

### Study area

The study was carried out in the Cape of Good Hope section of the Table Mountain National Park (34°16′S, 18°25′E), an area of 7 750 ha located about 60 km south of central Cape Town, South Africa. The predominant vegetation is fynbos, a fire-prone, sclerophyllous shrubland associated with nutrient-poor soils. The area experiences a mild Mediterranean-type climate: most of the ca 400–600 mm of annual rain falls in winter (May–Sept.), and maximum temperatures seldom exceed 30°C while minima of below 10°C are rarely recorded. On average, the fynbos in the park burns every 23 years, although the regime is quite variable with regards to frequency and season [43]. Of the 81 sites that we sampled, 6 (7.4%) burned three or more times, 51 (62.9%) burned twice, 15 (18.5%) burned once, and 9 (11.1%) did not burn at all. The local flora is extremely species-rich, containing 1 073 recorded species [44]. We regarded the entire study area as a meta-community [45]. This assumption is justified as a number of colonization events were observed, implying that patches (here represented by our sample sites) are connected by dispersal.

### Field methods

We re-located 81 of the 100, 5×10 m monitoring sites sampled by Hugh Taylor in 1966, by identifying the original site markers that demarcated the southwestern corner of each plot. Taylor's sites were located in a grid design that covered the entire study area. Sites that were not re-located either no longer had a visible permanent marker or had been destroyed by infrastructure development. Each of the re-located sites were re-sampled in 1996 by SDJ Privett with the occasional assistance of Taylor to ensure exact correspondence with the methods used during the 1966 survey. Accordingly, the 5×10 m sites were subdivided into ten subplots each of 2.5×2 m, which were examined individually in developing the species list for the site. Only perennially identifiable species were sampled (i.e. seasonally active geophytes and annuals were ignored). In each subplot, presence and number of individuals of every species was recorded at both dates. 422 species were recorded in 1966 and we recorded 396 in 1996. Approximately 10,000 individuals were recorded at both dates. Careful attention was paid to ensuring accurate identification and correct nomenclature in both the 1966 and 1996 data sets.

The site-abundance data are given in Appendix S1, with a clear explanation of the datasets.

Given the large flora sample in our study, we identified five easy-to-measure variables that were likely to explain the odds of local extinction and colonisation of different species. These were meta-community spread, meta-community species abundance, growth form, regeneration mode and dispersal distance. Meta-community spread was quantified for each species as the number of sites it occupied in 1966. Meta-community species abundance was calculated as the total number of individuals of each species in all sites in 1966. All species were characterised according to their growth form (Gf: : low shrub (<1 m), mid shrub (≥1 m but <2 m), tall shrub (≥2 m), graminoid, herb (excludes annuals), geophyte (apparent throughout the year)), regeneration mode (Reg: non-sprouter, sprouter) and dispersal distance (short distance (passive and ant), long distance (wind and bird)). The placement of species into these biological categories was based on published and personal observations.

### Extinction and colonisation measures

We described local extinction as the process whereby a population of a species disappeared from a site between the sampling times. The frequency of local extinction *f*(1,0) for each species present in 1966 was calculated as the number of sites in which the species occurred in 1966 but was no longer observed in 1996, divided by the number of sites in which the species occurred in 1966. Local colonisation was estimated in a similar fashion, replacing extinctions with colonisations.

### Statistical analyses

Our temporal study was restricted to a localised region containing a large number of closely related species. Taxonomic relatedness can often bias results by creating issues of autocorrelation [46], [47]. We thus used generalised linear mixed effects models (GLMM, [48]) with the five predictors as ‘fixed effects’, and the different levels of taxonomic relatedness (Class/Order/Family) as ‘random effects’, to reduce the influence of evolutionary history and avoid misinterpretation of the overall trend ([49]).

Stepwise regression-backward, forward or both–is an obvious method for examining the relative importance of each derived ecological variable to explain local extinctions and colonisations. Usually, stepwise regressions are based on the Akaike Information Criteria (AIC, [50]). In both backward and forward stepwise regressions, variables are tested sequentially, and the one producing the lowest AIC or BIC is retained. The method then assesses the contribution of the other variables after accounting for the selected one. This approach is appealing as it classifies the variables, ranks them based on their contribution to reducing the total AIC, and retains the most parsimonious combination of variables. However, using usual stepwise regression to find the optimal combination of explanatory variables to model a response is often considered to be a high-variance operation because small perturbations of the response data can sometimes lead to vastly different subsets of the variables [51], [52].

To avoid this problem, and to measure the actual power of each variable, we used multimodal inference based on all-subsets selection of generalised linear mixed effects models (GLMM) ([53]). This method has been proven to be more robust and useful than stepwise regression [53]–[55], and allows the measurement of the weight of evidence with which each explanatory variable explains the response variable [53]. In the case of five predictors, there are 2^5^ = 32 possible models in an all-subsets selection (e.g. four possible models with two predictors X1 and X2; M1: Y = 1; M2: Y = X1; M3: Y = X2; M4: Y = X1+X2). We thus estimated a small-sample (second order) bias adjustment of AIC (AICc) for each sub model. To estimate the weight of evidence of each predictor (w*_pi_*) to explain the frequency of extinction and colonisation, we simply summed the model AICs weights (w*_i_*) over all models in which predictor appeared.

To estimate the real power of our findings, we used a stratified permutation test [49]. This was created by random permutation of each predictor separately within the data set, re-calculating w*_pi_*, and repeating this procedure 100 times for each predictor. The absolute weight of evidence (Δw_p_) was then calculated by subtracting the median value of the 100 randomized w*_pi_* from the original w*_pi_*. Only predictors with Δw_p_ higher than zero have a certain explanatory power on local extinction or colonisation.

The estimation of the weight of evidence gives the absolute explanatory power of each predictor but does not provide predictions of extinction nor colonisation. To derive predicted local extinctions and colonisations, we averaged the predictions from each sub model weighted by the model AICc weight: 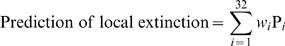, where P*_i_* is the prediction of sub model *i*, and w*_i_* is the absolute model *i* AICc weight. The prediction of local colonisation was extracted following the same procedure.

## Supporting Information

Appendix S1 Site-abundance dataset for the two sampling dates. The first two rows represent the sampling dates and the sites ID respectively. The species are stored by rows. Abundance was estimated in a way that was compatible with Taylor's method used in the 1966 survey. We counted the number of individuals of each species in a site and converted the data to a five-category system that corresponded to the abundance ratings used by Taylor (1984a). Thus, 1 = 1–4 individuals, 2 = 5–10, 3 = 11–50, 4 = 51–100, 5 = >100.
(0.54 MB XLS)
